# Voice Changes Related to Perceived Indoor Air Quality in Finnish Teachers: A Proof-of-Concept Field Study

**DOI:** 10.3390/ijerph23080973

**Published:** 2026-07-28

**Authors:** Hanna Vertanen-Greis, Sirkku Häkkilä, Eliisa Löyttyniemi, Juha Vintturi, Anthony Maalouf, Ahmed Geneid

**Affiliations:** 1 Faculty of Arts, Psychology and Theology, Speech and Language Pathology, Åbo Akademi University, 20500 Turku, Finland; 2 Aerobiology Laboratory, The Department of Biodiversity Sciences of the University of Turku, 20500 Turku, Finland; sirhak@utu.fi; 3 Department of Biostatistics, University of Turku and Turku University Hospital, 20500 Turku, Finland; eliisa.loyttyniemi@utu.fi; 4 Department of Otorhinolaryngology and Phoniatrics–Head and Neck Surgery, University of Helsinki and Helsinki University Hospital, 00290 Helsinki, Finlandanthony.maalouf@helsinki.fi (A.M.); ahmed.geneid@hus.fi (A.G.)

**Keywords:** voice, teacher, perceived indoor air quality, sick leave

## Abstract

**Highlights:**

**Public health relevance—How does this work relate to a public health issue?**

- Teachers commonly report voice symptoms linked to perceived indoor air quality.
- Workplace indoor environments are a key factor in occupational health.

**Public health significance—Why is this work of significance to public health?**

- Poorer perceived indoor air quality and longer exposure were associated with increased self-reported voice symptoms.
- No corresponding clinical or environmental associations were observed.

**Public health implications—What are the key implications or messages for practitioners, policy makers and/or researchers in public health?**

- Monitor and support teachers with persistent voice symptoms in occupational health care.
- Use longitudinal designs and real-time indoor air quality data to clarify causality.

**Abstract:**

This study examined associations between perceived indoor air quality (PIAQ) and self-reported voice symptoms in teachers, with exploratory observations on man-made mineral fibers (MMMF). Forty-seven teachers underwent within-day assessments at the beginning (good PIAQ) and end (poor PIAQ) of a workday. Clinical voice status was evaluated with laryngoscopy and the Grade–Roughness–Breathiness–Strain (GRBS) scale. Self-reported symptoms were measured with a 100 mm Visual Analog Scale (VAS) and the 30-item Voice Handicap Index (VHI). Classroom settled dust was collected on gelatin tape and analyzed by light microscopy for MMMF. No significant within-day clinical voice changes were detected, although breathiness showed a non-significant increase. Median VAS scores for voice symptoms showed no meaningful within-day change. Poorer afternoon PIAQ was associated with higher post-VAS, and longer daily exposure time co-varied with greater subjective worsening. Teachers with high VHI scores more often reported symptom worsening and poorer PIAQ ratings. Self-reported voice symptoms correlated positively with poor PIAQ and exposure duration. These findings suggest a symptom-driven pattern without evidence of environmental causality. Longitudinal studies with real-time IAQ monitoring are needed. Occupational health follow-up is recommended for teachers with persistent voice problems, with attention to vocal load.

## 1. Introduction

Voice disorders are common among teachers and have been associated with poor indoor environment quality [1,2]. Most previous research relies on self-reported symptoms and cross-sectional designs, whereas clinical investigations are rare [3].

Voice problems in teachers have been associated with increased sick leave [4,5]. Voice symptoms have also been associated with poor perceived indoor air quality (PIAQ), with frequent complaints including dryness, insufficient ventilation, and airborne irritants [6]. The perception of dry air is influenced not only by low humidity but also by air pollutants that impair the protective mucous layer in the airways, potentially leading to voice issues [7]. In our previous study [2], teachers with voice disorders reported more indoor environmental complaints, although objective measurements of air quality do not consistently correlate with clinical symptoms [8] and exposure may vary within buildings [9]. School environments pose challenges for maintaining air quality [10]. One potential irritant is man-made mineral fibers (MMMF), which are used for acoustic control. When damaged, these materials can release airborne fibers [11], potentially irritating airways at high concentrations [12]. As teachers often breathe through the mouth while speaking, inhaled particles may have a more direct pathway to the larynx.

Despite the central role of vocal health in teaching, clinical research examining voice changes in relation to indoor conditions remains limited. Thus, we investigated associations between voice changes and (1) PIAQ, (2) MMMF, and (3) background variables, explicitly framing this study as a proof-of-concept observational study of associations rather than causality due to the observational within-day design. This study focuses on perceived indoor air quality (PIAQ) and does not include objective IAQ measurements.

## 2. Materials and Methods

### 2.1. Study Design and Participants

**Figure 1:**
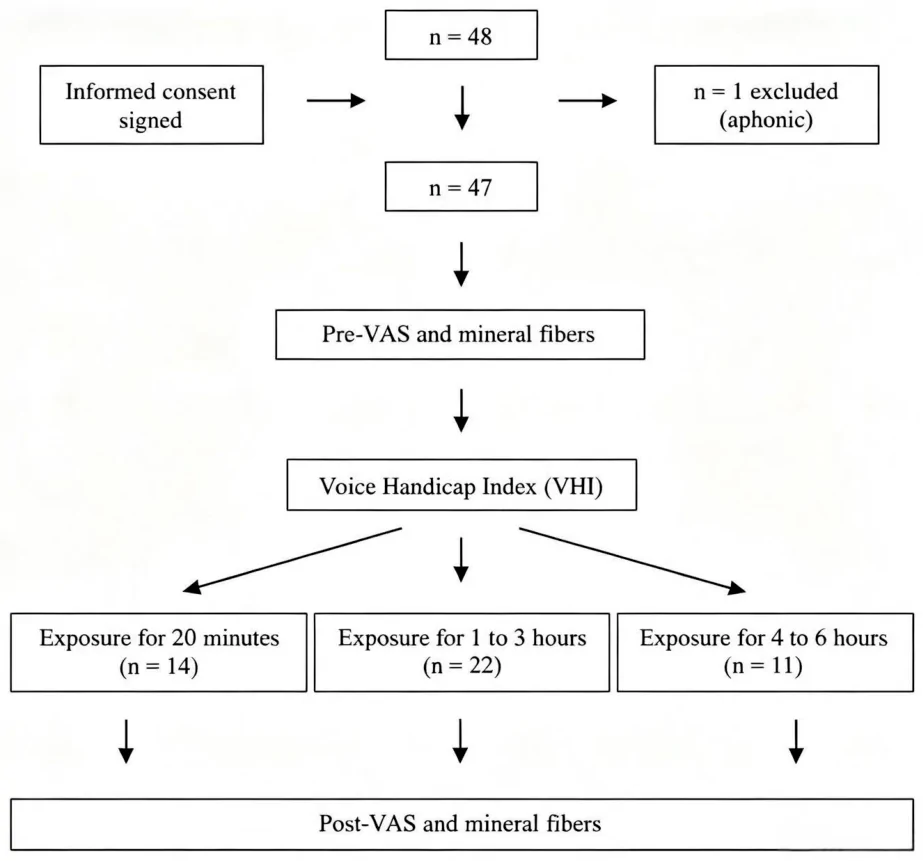
The study flow chart (*n* = 47).

To assess voice changes in environments perceived as having good versus poor PIAQ, we performed a field study among Finnish teachers in September 2017. We recruited participants from a prior survey (*n* = 1198 respondents) [2]. Participation in the clinical study was voluntary, and 47 teachers who met the inclusion criteria and were available on the examination day were included. All participants were actively working full-time teachers in Finnish schools. Laryngoscopy recordings of three participants were excluded because of poor visibility, although they remained in the study for the other assessments. The planned target sample size was 60, and 47 participated in the examination day due to absences and vacations. All participants provided written informed consent prior to participation. The study was conducted in accordance with the guidelines of the Declaration of Helsinki and was approved by the Ethics Committee of Helsinki University Hospital (1771/2017) and the University of Turku (26/2016). Figure 1 presents the study flow chart.

### 2.2. Clinical Voice Assessments

Indirect laryngoscopy was performed using a rigid endoscope (70°/90°, Olympus, Tokyo, Japan) and stroboscopic assessment during phonation using rpSzene^©^ (Rehder Partner GmbH, Hamburg, Germany). Four phoniatricians rated voices using the Grade–Roughness–Breathiness–Strain (*GRBS*) scale. The original GRBAS scale was modified by excluding the *asthenia* parameter, as none of the participants exhibited asthenic voice characteristics. Each *GRBS* parameter was rated on a four-point scale (normal, slight disorder, moderate, severe). Changes in *GRBS* scores (≥0.1 points) were categorized (decrease, no change, increase). Inter-rater agreement ranged from 36% to 60%, with disagreement between 11% and 34%.

### 2.3. Self-Reported Voice Assessments

Subjects completed a 100 mm Visual Analog Scale (*VAS*) with the statement “My voice is in poor condition,” ranging from “Not at all” (0 mm) to “Very much” (100 mm). Values ≤ 20 mm indicated good, 21–69 mm moderate, and ≥70 mm poor voice. We categorized *VAS* changes (voice getting worse, no change, voice getting better). We used the Voice Handicap Index (*VHI*) to classify perceived voice symptoms [13]. The 30 statements were rated on a 5-point Likert scale from “never” (0) to “always” (4). A *VHI* score of 0–30 indicates mild, 31–60 indicates moderate, and 61–120 indicates severe voice handicaps.

### 2.4. PIAQ, MMMF, and the Exposure Time

We selected two rooms per school based on teachers’ perceptions of indoor air quality. PIAQ is a subjective perceptual report and is not an objective exposure metric. Pre-assessments were conducted at the beginning of the workday in rooms perceived as good and post-assessments at the end of the workday in rooms perceived as poor. Each participant served as their own control for within-day comparisons across perceived conditions.

MMMF samples were collected from settled dust accumulated over a two-week period using gelatin tape. Sampling trays were placed at a height of 2 m in draft-free areas to allow dust to settle on Petri dishes. After one year of cold storage, the samples were analyzed at the Aerobiology Laboratory, University of Turku, and the fibers were counted using light microscopy. [14] *MMMF* levels were dichotomized as ‘No’ (≤0.2 fibers/cm^2^) and ‘Yes’ (>0.2 fibers/cm^2^) based on Finnish health regulations [15]. Because MMMF represents only settled dust, it does not quantify airborne exposure.

The study was conducted during a typical workday without interruption of lessons, so *exposure time* varied among participants. The analysis was categorized into three groups: (1) at least 20 min but less than one lesson (45 min, representing short exposure time), (2) 1–3 lessons (intermediate), and (3) 4–6 lessons (long). The 20 min threshold was chosen because vocal strain may develop rapidly.

### 2.5. Background Variables

Background variables included *sex*, *age*, *smoking* status (never-ex-current), and self-reported doctor-diagnosed *asthma* and *allergic rhinitis*. Employer records provided individual-level *sick leave* data for the past 12 months, including total, short-term (1–3 days, self-certified), and long-term (>3 days, medically certified) absences. Vocal load variables included *noise* (‘No’ [No–Somewhat–Cannot say] vs. ‘Yes’), maximum *group size*, weekly *number of lessons*, and *lessons before the post-assessment*. *Stress at work* was rated on a 5-point Likert scale and dichotomized as ‘No’ [No–Little–Somewhat] vs. ‘Yes’ [Rather–Very much] [16].

### 2.6. Statistical Analysis

*VAS change* was studied using the Wilcoxon signed-rank test. *VAS change* was not normally distributed, as validated with the Shapiro–Wilk test and by visual evaluation. The number of short-term *sick leaves* was compared with the pre-assessment *PIAQ* score using the Wilcoxon rank-sum test. The association between *VAS*, *VHI* and *MMMF* level categorization was analyzed using the Kruskal–Wallis test. The Kruskal–Wallis test was performed for the association between *VAS change* and categorized *exposure time,* continuing with Dwass–Steel–Critchlow–Fligner corrected pairwise comparisons. The association between categorized *VAS change* and categorized *exposure time* was assessed using Fisher’s exact test. The correlation between continuous *VHI* score (normally distributed) and *VAS* assessments, *VAS change*, and number of *sick leaves* was evaluated using Spearman’s correlation, while the association between *VHI* score and *age* was tested using Pearson’s correlation. The association between *VAS* score and *sex* was analyzed using a two-sample t-test, assuming unequal variances. GRBS data were treated as ordinal categories for descriptive change classifications. All statistical tests were performed using the significance level set at 0.05 (two-tailed). Analyses were performed using JMP^®^ 16 Pro for macOS (SAS Institute Inc., Cary, NC, USA) and SAS^®^ System, version 9.4 for Windows (SAS Institute Inc., Cary, NC, USA). All analyses were exploratory; no adjustment for multiple comparisons was applied. Reporting of this observational study follows the STROBE guideline; the completed STROBE checklist is provided in Supplementary File S1.

## 3. Results

The study sample consisted of 42 (89%) females and 5 (11%) males. The mean *age* was 44 years (range, 41–46 years) (Table 1). Teachers worked in 16 school buildings constructed between 1945 and 2006, most with centralized mechanical ventilation.

### 3.1. Clinical Voice Assessments

Laryngoscopy revealed anatomical findings in 5/47 (10.6%) participants (3 with vocal fold atrophy and 2 with bilateral vocal fold nodules). No anatomical or functional changes were observed between morning and afternoon. GRBS categories showed no significant changes, although we observed a trend of increased breathiness post-assessment (*p* = 0.089). In the pre-assessment, the *GRBS* scale showed a mean score of 0.655 (min–max; 0.0–2.0). In the post-assessment period, the mean was 0.590 (min–max; 0.1–2.2).

### 3.2. Self-Reported Voice Assessments

*VAS* ratings categorized 38% of the voices as good, 36% as moderate, and 26% as poor at baseline. Post-assessment, the distribution shifted to 32%, 43%, and 26%, respectively. Voice quality worsened in 36% of the participants, improved in 21%, and remained stable in 43%. The median *VAS* score remained unchanged at 40 mm (pre-assessment Q_1_–Q_3_, 14–70; post-assessment Q_1_–Q_3_, 20–77), resulting in a median *VAS change* of 0 mm (Q_1_–Q_3_, 0–12; *p* = 0.075).

*VHI* scores averaged 55 (range 31–100), indicating moderate voice handicap in 68% and severe voice handicap in 32%, as categorized. *VHI* score correlated with post-*VAS* (r_s_ = 0.45, *p* = 0.0014) and showed a positive but non-significant association with *VAS change* (r_s_ = 0.23, *p* = 0.11); higher VHI scores corresponded to higher post-VAS and the relationship with VAS change was suggestive but not significant. No associations emerged between anatomical findings and voice outcomes.

### 3.3. Voice Changes in Relation to PIAQ, MMMF, and Exposure Time

Altogether, 15 (32%) teachers were exposed for a short time (at least 20 min but less than one lesson), 21 (45%) teachers intermediately (1–3 lessons), and 11 (23%) for long (4–6 lessons). In the morning, only 7/47 (15%) teachers reported poor PIAQ, while in the afternoon, this increased to 33/47 (70%).

**Figure 2:**
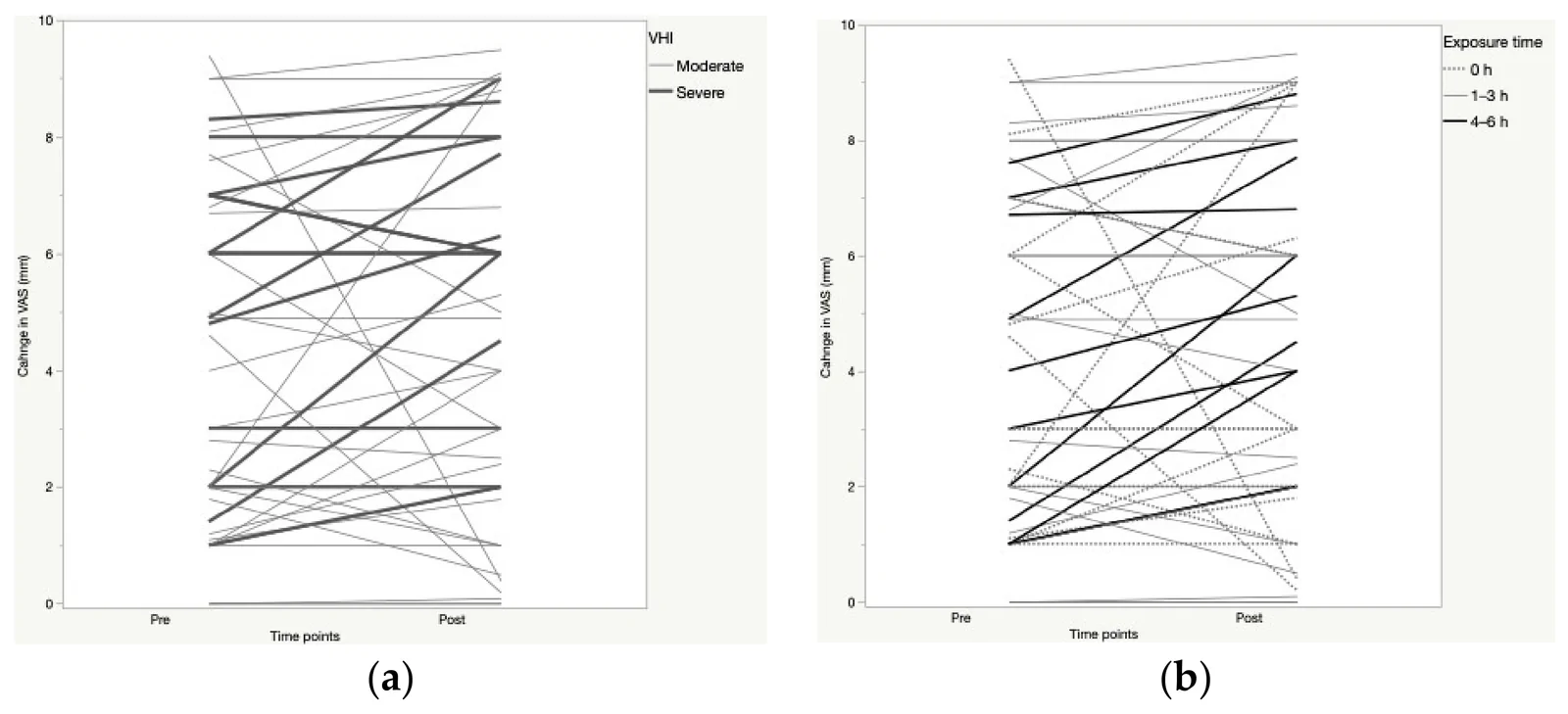
(**a**) Variability in individual changes in self-assessed voice using the Visual Analog Scale (VAS) between pre- and post-assessments in Finnish teachers (*n* = 47). The figure illustrates how each participant’s voice rating changed over time. (**b**) Change in self-assessed voice (VAS) by categorized exposure time (20 min–1 lesson, 1–3 lessons, 4–6 lessons).

**Figure 3:**
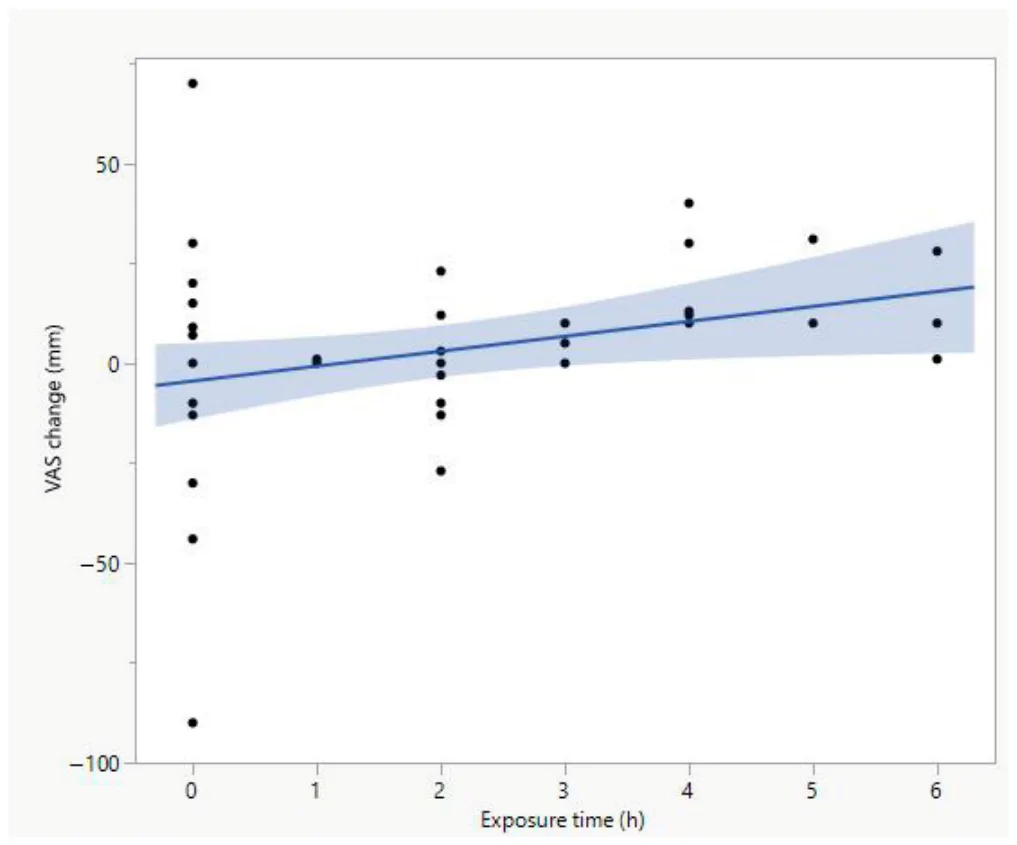
Association between exposure time (hours) and change in self-assessed voice using the Visual Analog Scale (VAS), with linear regression line, among Finnish teachers (*n* = 47).

Poor *PIAQ* was associated with poorer condition in voice (high post-*VAS* scores) (*p* = 0.0010). Longer exposure was associated with greater voice deterioration (VAS increase (*p* = 0.0010; Table 2). Nine of ten teachers with the long *exposure* reported voice deterioration, compared to one of four in the short *exposure* group and one of seven in the intermediate group (*p* = 0.0005; Table 3). Figure 2 illustrates the variability in individual *VAS change* between pre- and post-assessment, and Figure 3 shows the association between exposure time and VAS change, based on continuous exposure time (hours).

*MMMF* levels exceeded the threshold in 36% of classrooms considered good and in 21% considered poor. MMMF was analyzed as settled dust and showed no interpretable relationship with within-day voice outcomes.

### 3.4. Voice Changes in Relation to Background Variables

Males reported lower post-VAS scores than females (*p* < 0.0001). *VHI* score was associated positively with total and long-term *sick leave* (Table 4). Teachers with severe voice handicap had a median of 13 *sick leave* days (Q_1_ = 6; Q_3_ = 27), compared to a median of 5 days among those with moderate voice handicap (Q_1_ = 2; Q_3_ = 15.5). Teachers who perceived poor *PIAQ* had significantly more short-term sick leave (*p* = 0.038). Noise exposure was associated with higher *VHI* scores. No other associations were found between pre-*VAS* or *VAS change* and the background variables. Smokers showed numerically greater VAS worsening, but differences were not statistically significant due to small sample size (*n* = 4).

## 4. Discussion

This proof-of-concept study reports associations between PIAQ and self-reported voice symptoms, avoiding environmental causal language. No clinical voice changes were found between classrooms classified as having good or poor PIAQ. However, teachers who reported voice symptoms experienced more voice strain during the workday and had more sick leave than others. Although this may seem contradictory, it reflects a well-documented phenomenon: subjective symptoms and poor PIAQ often co-occur even when objective measurements fail to confirm measurable changes. Teachers with voice symptoms more often perceived air quality as poor, but these perceptions were not supported by voice changes or by MMMF. This study focuses on perceived indoor air quality (PIAQ) and does not include objective IAQ measurements.

### 4.1. Clinical and Self-Reported Voice Assessments

We found no clinical voice changes between the two time points, consistent with earlier findings that reported only minor, non-significant changes after prolonged voice use [17]. Other studies have observed clinical or mild changes in a notable proportion of teachers even without associations with self-reported symptoms [18]. These results suggest that objective and subjective voice assessments may not always align. Importantly, because the morning and afternoon assessments were conducted in different classrooms and at different times of day, the study design does not allow separation of perceived environmental influences from cumulative vocal loading or time-dependent vocal fatigue. Consequently, any interpretation of symptom differences must be made cautiously and should not be attributed specifically to IAQ. Within this study, between-group differences by exposure time aligned with symptom reports, while overall pre-to-post clinical change did not emerge. Teachers with perceived voice symptoms experienced greater deterioration in voice quality during the workday and reported more sick leave than their colleagues did. Our findings are consistent with those of previous studies showing that vocal fatigue is associated with increased sick leave among teachers [5]. Taken together, the pattern is compatible with symptom-driven perception. The relatively modest inter-rater agreement of the GRBS assessments should also be considered when interpreting the absence of clinical changes. Although no systematic within-day changes were detected, limited agreement among raters may have reduced sensitivity to subtle perceptual voice differences. Therefore, subtle clinical changes cannot be entirely excluded.

### 4.2. Voice Changes, PIAQ, MMMF, and Exposure Time

The worse the teachers scored their voice, the worse they perceived IAQ. In contrast, those with good voice scores did not report poor air quality. These findings are consistent with those of earlier studies that demonstrated an association between perceived voice symptoms and poor indoor environments. Prior research has shown that both subjective and objective assessments of indoor air quality can be associated with voice-related symptoms [5].

As shown in the results, self-reported voice symptoms increased significantly in the long exposure group. This finding is particularly noteworthy, although the small group size (*n* = 11) may have amplified the effect. Nevertheless, the data indicate that longer exposure co-occurred with greater symptom reporting. These findings are consistent with previous voice research showing that prolonged voice use is associated with increasing vocal fatigue, throat discomfort, and perceived deterioration of voice quality during the workday [19,20]. Although our study did not directly quantify vocal loading, the exposure-time pattern observed in Table 3 supports the possibility that cumulative voice use contributed to the reported symptoms. Although overall median VAS did not change, exposure-time groups differed, indicating symptom variation rather than uniform within-day effects.

MMMF was assessed from settled dust accumulated over a two-week period, reflecting material deposited from indoor air over time rather than contemporaneous inhalation exposure at the time of assessment. As MMMF is therefore not a real-time exposure measure, no inference regarding same-day inhalation exposure can be made. *MMMF* was detected in several classrooms, but no significant associations were observed with voice symptoms or sick leave. Settled-dust MMMF does not quantify airborne exposure and cannot be temporally aligned with within-day symptom changes. Previous studies have reported mixed results regarding *MMMF* [11,21]. Given the limited evidence linking IAQ and MMMF to voice symptoms, vocal load is a plausible contributing factor. However, causality cannot be inferred, and vocal load could not be disentangled from perceived environmental factors. Teachers are exposed to prolonged voice use, and previous research has consistently linked vocal load with voice disorders. Teaching load aligned more consistently with symptoms than environmental measures. These findings do not support an MMMF effect on same-day voice outcomes. Findings from continuous exposure analysis (Figure 3) further support a positive association between exposure duration and VAS change.

### 4.3. Background Variables

We found no significant associations between pre-*VAS* or *VAS change* and *asthma*, *allergic rhinitis*, *stress at work*, or *smoking*. However, males reported better post-*VAS* scores than females although caution is warranted due to the small male sample (*n* = 5). Teachers reporting *noise* exposure had higher *VHI* scores than those who did not, consistent with earlier findings [22]. Although we found no significant associations between *VAS changes* and *group size* or weekly *number of lessons*, a higher number of lessons before the post-assessment was associated with worsening voice symptoms, consistent with cumulative voice use. Because no objective signs of poor indoor conditions were found, teaching load aligned more closely with symptom changes. This aligns with prior evidence that extensive vocal use increases the risk of voice disorders [1], although assessing the precise impact of vocal load remains methodologically challenging [19]. Experimental work shows that throat discomfort and fatigue increase with continued voice use [20].

Teachers who perceived poor PIAQ before exposure had more short-term sick leave, and those with severe voice handicap had more absence days than those with moderate handicap. High VHI scores were also associated with increased long-term sick leave. This finding is in agreement with earlier studies reporting that teachers with more severe voice-related difficulties experience higher levels of work impairment and absence from work [4,5]. In our study, teachers with severe voice handicap reported substantially more sick leave than teachers with moderate voice handicap. The association observed in Table 4 therefore supports the clinical relevance of self-perceived voice handicap even in the absence of corresponding objective voice findings. These findings are consistent with earlier studies reporting increased sick leave among teachers with voice problems [4], and align with national data on Finnish teachers [23]. Longer exposure during the day was likewise associated with greater voice deterioration.

### 4.4. Strengths and Limitations

Strengths included the within-subject design, use of validated tools, and employer-verified sick leave data. The sample size was small (*n* = 47), and several subgroup analyses were based on very small groups, particularly the long-exposure category (*n* = 11). Consequently, statistical precision was limited and some observed effects may have been unstable. However, the study was designed as a proof-of-concept field investigation intended to explore potential associations and generate hypotheses for future larger studies. The sample was nevertheless demographically comparable with Finnish teacher populations reported previously [23]. PIAQ was explicitly treated as perception rather than exposure. Because the term “poor indoor environment” was not precisely defined, interpretations may have varied. Self-reported data are possible limitations. In addition, participants were recruited from a previous survey cohort and participation in the clinical study was voluntary. Consequently, teachers who chose to participate may have been more aware of voice symptoms or indoor environmental concerns than non-participants. This potential selection bias may have influenced the observed associations and may limit generalizability of the findings. However, these factors are unlikely to fully account for the observed associations [24]. The VAS item may have primed negative responses. We could not control the exposure time or separate the effects of vocal load from indoor air quality. Voices typically recover between days, limiting the value of extended follow-up for within-day patterns. Long-term effects remain possible given prolonged work in these environments, although the study focused on same-day observations apart from MMMF sampling with the focus on subjective experience except for the MMMF measurements. This study did not assess potential effects on students; future studies should evaluate whether similar symptom patterns are observed among pupils.

## 5. Conclusions

Self-reported voice symptoms co-varied with poor PIAQ and longer exposure time, while clinical assessments and MMMF showed no associations. Future research should include objective IAQ logging and ecological momentary symptom assessment. Teachers who reported voice symptoms also showed higher VAS scores at the end of the workday. They also reported more sick leave and poorer PIAQ. Occupational health services should monitor teachers with persistent voice problems and consider vocal load assessment rather than environmental remediation alone.

## Figures and Tables

**Table 1:** Demographic characteristics of the Finnish teacher study sample (*n* = 47).

Demographic Characteristic			
Sex, *n* (%)	Female	42	(89)
	Male	5	(11)
Age, Mean (min–max)		44	(41–46)
Smoking, *n* (%)	Never smoked	30	(64)
	Ex-smoker	13	(28)
	Current smoking	4	(8)
Asthma, *n* (%)		12	(26)
Allergic rhinitis, *n* (%)		18	(39)
Sick leaves, Median (Q_1_–Q_3_)	Total number of days of sick leaves	8	(2–20)
	Number of short-term sick leaves	3	(1–5)
	Number of long-term sick leaves	0	(0–1)
Noise, *n* (%)		17	(35)
Group size (max), Median (Q_1_–Q_3_)		22	(18–25)
Lessons (45 min) per week, Median (Q_1_–Q_3_)		26	(24–28)
Lessons (45 min) before post-assessment, Median (Q_1_–Q_3_)		4	(2–5)
Stress at work, *n* (%)		7	(15)

**Table 2:** Association between changes in voice symptoms (measured using the Visual Analog Scale, VAS) and exposure time among Finnish teachers (*n* = 47). Overall test result was *p* = 0.0010, indicating statistical significance; therefore, pairwise comparisons were conducted. Significant *p* values are indicated with an asterisk (*).

Exposure Time	Change in VAS, Median	(95% CI)	*p* Value
20 min–1 lesson (short)	0	(−1.3–1.5)	20 min vs. 1–3 h 0.97
1–3 lessons (intermediate)	0	(−0.3–0.1)	20 min vs. 4–6 h 0.041 *
4–6 lessons (long)	1.2	(1.0–3.1)	1–3 h vs. 4–6 h 0.0003 *

**Table 3:** Association between changes in voice symptoms (measured using the Visual Analog Scale, VAS) and exposure time among Finnish teachers (*n* = 47; *p* = 0.0005).

Exposure Time	Voice Getting Worse		No Change		Voice Getting Better		Total *n*
	*n*	(%)	*n*	(%)	*n*	(%)	
20 min–1 lesson (short)	4	(26.7)	6	(40.0)	5	(33.3)	15
1–3 lessons (intermediate)	3	(14.3)	13	(61.9)	5	(23.8)	21
4–6 lessons (long)	10	(90.9)	1	(9.1)	0	(0.0)	11

**Table 4:** Associations between voice assessments using Voice Handicap Index (VHI) and background variables in Finnish teachers (*n* = 47). Significant *p* values are indicated with an asterisk (*).

Variable		VHI Score, Mean/ρ ^1^/r_s_^2^	(95% CI)	*p* Value
Sex	Female	56	(51–61)	0.082
	Male	43	(31–55)	
Age		0.28 ^1^	(−0.0124–0.52)	0.0606
Asthma	No	54	(49–58)	0.52
	Yes	57	(44–70)	
Allergic rhinitis	No	52	(46–58)	0.17
	Yes	58	(51–66)	
Noise	No	51	(46–56)	0.015 *
	Yes	62	(53–71)	
Stress at work	No	53	(48–58)	0.15
	Yes	62	(45–80)	
Sick leaves	Total number of days of sick leaves	0.34 ^2^		0.021 *
	Number of short-term sick leaves	0.11 ^2^		0.45
	Number of long-term sick leaves	0.41 ^2^		0.0041 *

^1^ Pearson correlation. ^2^ Spearman’s correlation. Abbreviations: CI: confidence interval.

## Data Availability

The data presented in this study are available at https://data.mendeley.com/drafts/mgg6g9b9zn, accessed on 7 April 2022.

## Supplementary Materials

The following supporting information can be downloaded at: https://www.mdpi.com/article/10.3390/ijerph23080973/s1, File S1: STROBE checklist.

## Abbreviations

The following abbreviations are used in this manuscript:

PIAQ	Perceived indoor air quality
MMMF	Man-made mineral fibers
GRBS	Grade–Roughness–Breathiness–Strain
VAS	Visual Analog Scale
VHI	Voice Handicap Index

## References

[1] Cantor Cutiva L.C., Vogel I., Burdorf A. (2013). Voice disorders in teachers and their associations with work-related factors: A systematic review. J. Commun. Disord..

[2] Vertanen-Greis H., Löyttyniemi E., Uitti J., Putus T. (2023). Self-reported voice disorders of teachers and indoor air quality in schools: A cross-sectional study in Finland. Logop. Phoniatr. Vocol.

[3] Wolkoff P. (2018). Indoor air humidity, air quality, and health: An overview. Int. J. Hyg. Environ. Health.

[4] de Medeiros A.M., Assuncao A.Á., Barreto S.M. (2012). Absenteeism due to voice disorders in female teachers: A public health problem. Int. Arch. Occup. Environ. Health.

[5] Lyberg Åhlander V., Rydell R., Löfqvist A. (2011). Speaker’s comfort in teaching environments: Voice problems in Swedish teaching staff. J. Voice.

[6] Korn G.P., Augusto de Lima Pontes A., Abranches D., Augusto de Lima Pontes P. (2015). Hoarseness and risk factors in university teachers. J. Voice.

[7] Geneid A., Ronkko M., Airaksinen L., Voutilainen R., Toskala E., Alku P., Vilkman E. (2009). Pilot study on acute voice and throat symptoms related to exposure to organic dust: Preliminary findings from a provocation test. Logop. Phoniatr. Vocol.

[8] Sliwinska-Kowalska M., Niebudek-Bogusz E., Fiszer M., Los-Spychalska T., Kotylo P., Sznurowska-Przygocka B., Modrzewska M. (2006). The prevalence and risk factors for occupational voice disorders in teachers. Folia Phoniatr. Logop..

[9] Bakke J.V., Norback D., Wieslander G., Hollund B.E., Florvaag E., Haugen E.N., Moen B.E. (2008). Symptoms, complaints, ocular and nasal physiological signs in university staff in relation to indoor environment: Temperature and gender interactions. Indoor Air.

[10] Bayer C.W., Crow S.A., Fischer J. (2000). Causes of Indoor Air Quality Problems in Schools. Summary of Scientific Research.

[11] Tuomi T., Wallenius K., Mahiout S., Rautiala S., Lappalainen S. (2020). Teolliset Mineraalikuidut Toimistotyyppisissä Työtiloissa: Esiintyminen, Altistumisen Arviointi, Terveysvaikutukset ja Päästöjen Hallinta [Man-Made Vitreous Fibers in Office Environment].

[12] ATSDR (2004). Toxicological Profile for Synthetic Vitreous Fibers.

[13] Jacobson B.H., Johnson A., Grywalski C., Silbergleit A., Jacobson G., Benninger M.S., Newman C.W. (1997). The Voice Handicap Index (VHI): Development and validation. Am. J. Speech Lang. Pathol..

[14] (2017). Kuitunäytteen Ottaminen Teippimenetelmällä [Instructions for Fiber Sampling by Tape Method].

[15] (2015). Asumisterveysasetus [Decree of the Ministry of Social Affairs and Health on Health-Related Conditions of Housing and Other Residential Buildings and Qualification].

[16] Elo A.L., Leppänen A., Jahkola A. (2003). Validity of a single-item measure of stress symptoms. Scand. J. Work Environ. Health.

[17] Pellicani A.D., Fontes A.R., Santos F.F., Pellicani A.D., Aguiar-Ricz L.N. (2018). Fundamental Frequency and Formants Before and After Prolonged Voice Use in Teachers. J. Voice.

[18] Ilomäki I., Leppanen K., Kleemola L., Tyrmi J., Laukkanen A.M., Vilkman E. (2009). Relationships between self-evaluations of voice and working conditions, background factors, and phoniatric findings in female teachers. Logop. Phoniatr. Vocol.

[19] Laukkanen A.M., Ilomäki I., Leppanen K., Vilkman E. (2008). Acoustic measures and self-reports of vocal fatigue by female teachers. J. Voice.

[20] Vintturi J., Alku P., Sala E., Sihvo M., Vilkman E. (2003). Loading-related subjective symptoms during a vocal loading test with special reference to gender and some ergonomic factors. Folia Phoniatr. Logop..

[21] Tähtinen K., Lappalainen S., Karvala K., Lahtinen M., Salonen H. (2019). Probability of Abnormal Indoor Air Exposure Categories Compared with Occupants’ Symptoms, Health Information, and Psychosocial Work Environment. Appl. Sci..

[22] Martins R.H.G., Pereira E.R.B.N., Hidalgo C.B., Tavares E.L.M. (2014). Voice disorders in teachers. A review. J. Voice.

[23] The Finnish Public Sector Study. https://www.ttl.fi/en/research/projects/finnish-public-sector-study-fps.

[24] Fisk W.J., Lei-Gomez Q., Mendell M.J. (2007). Meta-analyses of the associations of respiratory health effects with dampness and mold in homes. Indoor Air.

